# Loss of mesenchymal bone morphogenetic protein signaling leads to development of reactive stroma and initiation of the gastric neoplastic cascade

**DOI:** 10.1038/srep32759

**Published:** 2016-09-09

**Authors:** Sébastien A. B. Roy, Joannie M. Allaire, Camille Ouellet, Faiza Maloum-Rami, Véronique Pomerleau, Étienne Lemieux, Jean-Philippe Babeu, Jasmin Rousseau, Marilène Paquet, Perrine Garde-Granger, François Boudreau, Nathalie Perreault

**Affiliations:** 1 Département d’Anatomie et Biologie Cellulaire, Faculté de Médecine et des Sciences de la Santé, Université de Sherbrooke, Sherbrooke, QC, Canada; 2 Département de pathologie et de microbiologie, Faculté de Médecine Vétérinaire, Université de Montréal, St-Hyacinthe, QC, Canada; 3 Département de Pathologie, Faculté de Médecine et des Sciences de la Santé, Université de Sherbrooke, Sherbrooke, QC, Canada

## Abstract

Bmps are morphogens involved in various gastric cellular functions. Studies in genetically-modified mice have shown that Bmp disruption in gastric epithelial and stromal cell compartments leads to the development of tumorigenesis. Our studies have demonstrated that abrogation of gastric epithelial Bmp signaling alone was not sufficient to recapitulate the neoplastic features associated with total gastric loss of Bmp signaling. Thus, epithelial Bmp signaling does not appear to be a key player in gastric tumorigenesis initiation. These observations suggest a greater role for stromal Bmp signaling in gastric polyposis initiation. In order to identify the specific roles played by mesenchymal Bmp signaling in gastric homeostasis, we generated a mouse model with abrogation of Bmp signaling exclusively in the gastro-intestinal mesenchyme (*Bmpr1a*^ΔMES^). We were able to expose an unsuspected role for Bmp loss of signaling in leading normal gastric mesenchyme to adapt into reactive mesenchyme. An increase in the population of activated-fibroblasts, suggesting mesenchymal transdifferentiation, was observed in mutant stomach. *Bmpr1a*^*Δ*MES^ stomachs exhibited spontaneous benign polyps with presence of both intestinal metaplasia and spasmolytic-polypeptide-expressing metaplasia as early as 90 days postnatal. These results support the novel concept that loss of mesenchymal Bmp signaling cascade acts as a trigger in gastric polyposis initiation.

The mouse stomach is subdivided into 3 regions with very distinct histological features. The most proximal third, the forestomach, is lined with a stratified squamous epithelium. The corpus is the middle region and the distal region is the pyloric antrum. Both regions are composed of mucosal glands lined by a simple columnar epithelium^1^. The mucosal gland is divided into four regions, namely pit, isthmus, neck and base and is simultaneously composed of undifferentiated pluripotent stem cells and differentiated functional epithelial cells. Pluripotent stem cells and undifferentiated progenitor cells are located in the isthmus of the gland, and mitotic activity is confined to this area. The pit is composed of surface mucous secreting cells whereas mucous neck cells are found in the neck region and the glandular base contain the pepsinogen-secreting zymogenic cells. Acid-secreting parietal cells are found scattered throughout the lower two third of the mucosal gland. Parietal and zymogenic cells are absent from the pyloric antrum, which is mostly composed of surface mucous cells^2^,^3^.

The functional complexity of a multicellular organ such as the stomach involves a high degree of coordination among diverse specialized cell types. Maintaining this organization and regulation of gastric cell functions requires a dynamic crosstalk between epithelial and underlying stromal cells^4^,^5^,^6^,^7^,^8^. The normal gastric stroma consists of an integrated community of professional and non-professional cells^9^. Epithelial-mesenchymal interactions are decisive for stomach morphogenesis^4^,^5^,^10^ and functional maintenance of the glandular axis^5^. These interactions involve a specific type of stromal non-professional cells located beneath the epithelial cells in the *lamina propria*, designated as activated-fibroblasts or myofibroblasts. Activated-fibroblasts can arise from multiple origins, such as mesenchymal stem cells, fibrocytes or bone marrow-derived cells^11^,^12^,^13^. Under normal conditions, activated-fibroblasts mostly stem from the differentiation of resident fibroblasts^12^. These cells express both vimentin and smooth-muscle actin (α-Sma) and secrete numerous cytokines, growth factors (Wnt, FGFs and Bmps) and extracellular matrix (ECM) proteins, all of which contribute extensively to the establishment of the extracellular milieu that will ultimately become the microenvironment supporting and instructing the epithelial cells during processes ranging from embryogenesis, adult homeostasis and pathogenesis^7^,^9^,^14^,^15^.

Bone morphogenetic proteins (Bmps) are growth factors belonging to the TGF-β superfamily and play active roles in a number of developmental processes as well as in various cellular functions^16^. Bmp ligands and their receptors are expressed in both epithelial and mesenchymal compartments of the gut^6^,^17^,^18^. Thus, Bmps are not only able to act specifically on the epithelial compartment but also activate a signaling cascade within mesenchymal cells^7^,^8^,^18^. This mesenchymal signaling influences the microenvironment in acquiring a specific identity that will subsequently impact epithelial functions through epithelial-mesenchymal interaction^7^,^18^,^19^. Mutations affecting Bmp signaling are frequently observed in juvenile polyposis syndrome (JPS)^20^,^21^ as well as in tumor progression in the stomach^22^,^23^. Overexpansion of the mesenchymal compartment is observed in JPS hamartomas suggesting a role for the mesenchyme in their development^20^,^21^. Previous studies using genetic approaches in mice have revealed a role for Bmp signaling in gastric tumorigenesis^24^,^25^,^26^,^27^,^28^. These various mouse models included a broad range of Bmp signaling deletions in which signaling was lost in all cell layers of the tissue in presence or not of an inflammatory trigger. Conversely, studies from our laboratory, using the *Foxa3* promoter as the driver for Cre expression, revealed that exclusive loss of gastric Bmp signaling in the epithelium was not sufficient to trigger gastric tumorigenesis^6^, indicating that abrogation of epithelial Bmps signaling in the stomach alone was not sufficient to recapitulate the neoplastic features associated with total gastric loss of Bmp signaling^24^,^25^,^27^,^29^. These findings support the premise that Bmps signaling originating from the epithelium and the mesenchyme differentially regulate certain behaviors and functions of gastric epithelial cells.

The vast majority of gastric tumors arise in the epithelium, giving rise to adenocarcinomas. Alterations in a number of tumor suppressors, including *p53*, *E-cadherin* and *p16*, and oncogenes, such as β-*catenin* and *K-ras*, have been linked to gastric carcinogenesis, but most of these are found in only a fraction of gastric cancers^30^. Inflammation also plays a key role in the development of gastric cancer which is strongly linked to infection with *H. pylori*^30^. So far, most studies on gastric tumorigenesis have focused on identifying, within the epithelial compartment, the factors and signaling pathways affecting the proliferation of epithelial cells. GI cancer should no longer be regarded only as an epithelial problem where an accumulation of somatic mutations will result in uncontrolled growth. It is now well established that genetic changes in tumor epithelium are insufficient to fully explain tumor initiation, progression and metastasis^31^,^32^,^33^. The cancerous reactive mesenchyme observed in the pathology is frequently populated by an abnormal number of activated-fibroblasts. This reactive mesenchyme lead to the production of a toxic microenvironment for the epithelium with deregulated levels or types of cytokines, growth factors and a massive remodeling of the ECM, which can promote tumor initiation or its progression^14^,^33^,^34^. However, the mechanisms, signaling pathways and/or genetic mutations by which normal gastric mesenchyme become reprogrammed to become reactive is not well defined. Since epithelial deletion of Bmp signaling is not sufficient to induce gastric tumor formation, we developed a genetically-modified mouse model to inactivate the *Bmp type 1a receptor* (*Bmpr1a*) specifically in the gastrointestinal sub-epithelial mesenchyme in order to specify the function of the mesenchymal Bmp signaling cascade in gastric tumorigenesis initiation. Our results suggest an involvement for Bmp signaling on the commitment and determination of gastric sub-epithelial mesenchymal cells toward an activated-fibroblasts phenotype. Using this model, we have uncovered that mesenchymal Bmp signaling is essential for maintaining a healthy microenvironment supporting gastric epithelial cells. Indeed, loss of Bmp signaling in gastric sub-epithelial mesenchyme leads to the development of an overreactive mesenchyme generating a toxic microenvironment for the epithelium, hence promoting gastric polyposis initiation.

## Results

### Loss of gastric mesenchymal Bmpr1a leads to epithelial hyperplasia, disturbed glandular architecture and polyposis

**Figure 1:**
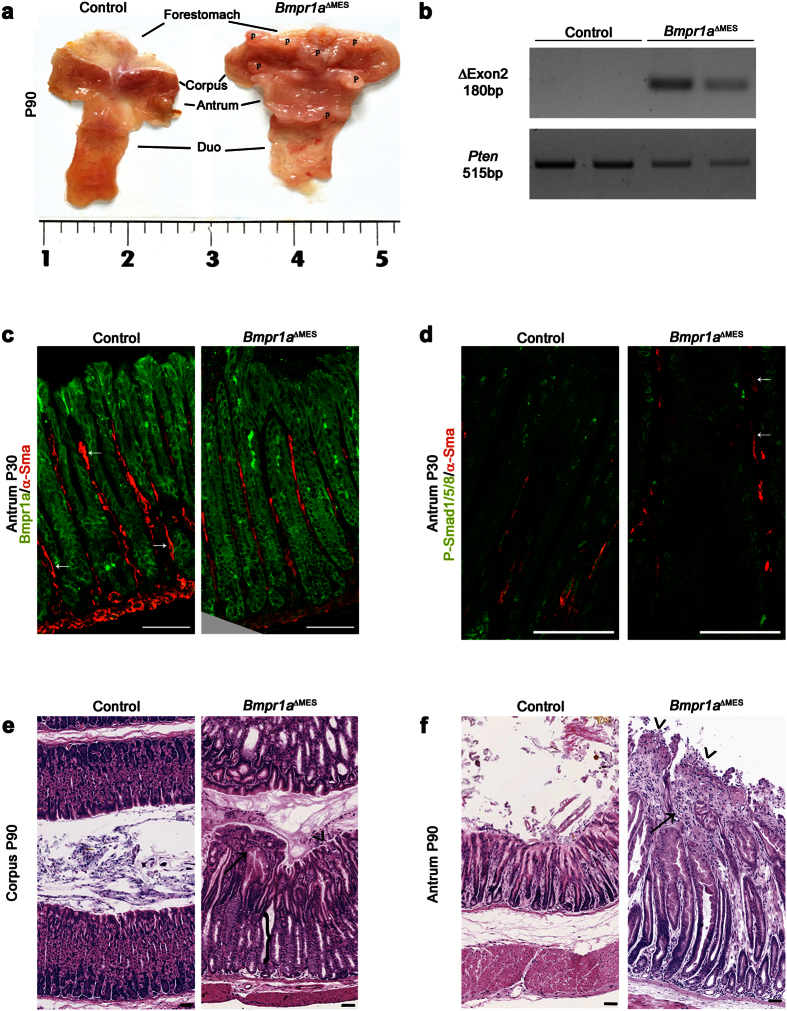
Loss of Bmp signaling in gastric activated-fibroblasts leads to the development of polyps and deregulates gastric glandular epithelial architecture. (**a**) Stomach macroscopic analysis revealed the presence of benign gastric polyps at 90 days of age in both the corpus and pyloric antrum in mutant mice compared to none in control mice. (**b**) *In vivo* Cre-dependent recombination of the loxP allele was validated using PCR amplification with specific primers on genomic DNA from mesenchymal gastric fractions of 1-year-old mice. The amplified 180-bp fragment corresponding to Cre-dependent recombination is observed in mesenchymal fractions of *Bmpr1a*^ΔMES^ mice (n = 2). PCR amplification with *Pten* specific primers on genomic DNA served as an integrity control. (**c**) Co-staining (white arrows) with α-Sma (red staining) and Bmpr1a (green staining) revealed a decrease in double-positive activated-fibroblasts adjacent to the epithelium in 30 days-old *Bmpr1a*^ΔMES^ antrum compared to controls. (**d**) Co-staining with α–Sma (red staining) and pSmad1-5-8 (green staining) revealed positive activation of the Bmp signaling pathway in stromal cells of 30-day-old control antrum. Co-staining in mutant mice revealed a decrease in double-positive myofibroblast (white arrows) cells in *Bmpr1a*^ΔMES^ mice compared to controls. (**e**) H&E staining performed on the stomach of 90-day-old control and mutant mice revealed significant perturbation of the gastric glandular architecture in mutant corpus regions as well as in (**f**) the pyloric antrum. Corpus gastric glands of *Bmpr1a*^ΔMES^ mice displayed abnormal morphology with decrease in parietal cell population (e, black bracket). Both corpus and pyloric gastric glands of *Bmpr1a*^ΔMES^ mice displayed abnormal morphology with gland fusion, vacuolization, immune cell infiltration (black arrow) and surface erosion (black arrowhead). Scale bar: 50 μm. Two-way ANOVA. **** *p* < 0.0001. P, polyps; Duo, duodenum.

Mice homozygous for the floxed exon 2 of the *Bmpr1a* gene^35^ were crossbred with the *Foxl1*Cre line, which directs Cre expression at E8.5 exclusively in the non-professional sub-epithelial mesenchymal cells population of the GI tract as demonstated by Sackett *et al*.^36^. Conditional knockout mice for *Bmpr1a* (*Bmpr1a*^ΔMES^) were born at the expected Mendelian ratios without obvious gross physical abnormalities. Macroscopic analysis of the stomach revealed the presence of gastric polyps in 90-day-old mutants compared to none in controls (Fig. 1a). PCR analysis was performed on genomic DNA obtained from MatriSperse-enriched mesenchymal gastric fractions of adult mice (Fig. 1b). These results confirmed that the recombination of the *Bmpr1a* floxed alleles occured in the mesenchymal fraction of the *Bmpr1a*^ΔMES^ mice (Fig. 1b). Loss of Bmpr1a expression exclusively in sub-epithelial mesenchyme was assessed by immunofluorescence. Bmpr1a immunostaining in control antrum was observed in both gastric epithelial and mesenchymal compartments (Fig. 1c). Co-immunostaining with α-Sma antibody allowed better visualization of activated-fibroblasts adjacent to the epithelium. In comparison to controls, immunoreactivity to Bmpr1a in 30-day-old mutant antrum was exclusively lost in mesenchyme cells adjacent to the epithelium (Fig. 1c). Given that phosphorylation and nuclear translocation of the Bmp effectors Smad-1-5-8 are functional features of an active Bmp signaling pathway^16^, loss of Bmp signaling exclusively in activated-fibroblasts was assessed by co-immunofluorescence of phosphorylated-Smad-1-5-8 (pSmad-1-5-8) and α-Sma. At 30 days, pSmad-1-5-8 nuclear immunostaining in control mice was observed in both gastric epithelial and mesenchymal compartments (Fig. 1d) while immunoreactivity to pSmad-1-5-8 in *Bmpr1a*^ΔMES^ mice was exclusively lost in α-Sma positive cells in *Bmpr1a*^ΔMES^ mice as opposed to gastric epithelial cells (Fig. 1d). H&E staining demonstrated an important thickening of the glandular mucosa and an abnormal epithelial glandular architecture in 90-days-old mutants (Fig. 1e,f). An enlargement of the mesenchymal compartment was also noted in the oxyntic glands (Fig. 1e) and pyloric antrum (Fig. 1f) of *Bmpr1a*^ΔMES^ mice relative to controls. Histopathological analysis revealed the presence of atypical and diffuse multifocal hyperplasia with surface erosions accompanied by necrosis of the gastric mucosa in the stomachs of *Bmpr1a*^ΔMES^ mice compared to controls (Fig. 1e,f). Presence of inflammatory infiltrates and fibrosis was observed in the enlarged mesenchymal compartment of the mutant stomach.

**Figure 2:**
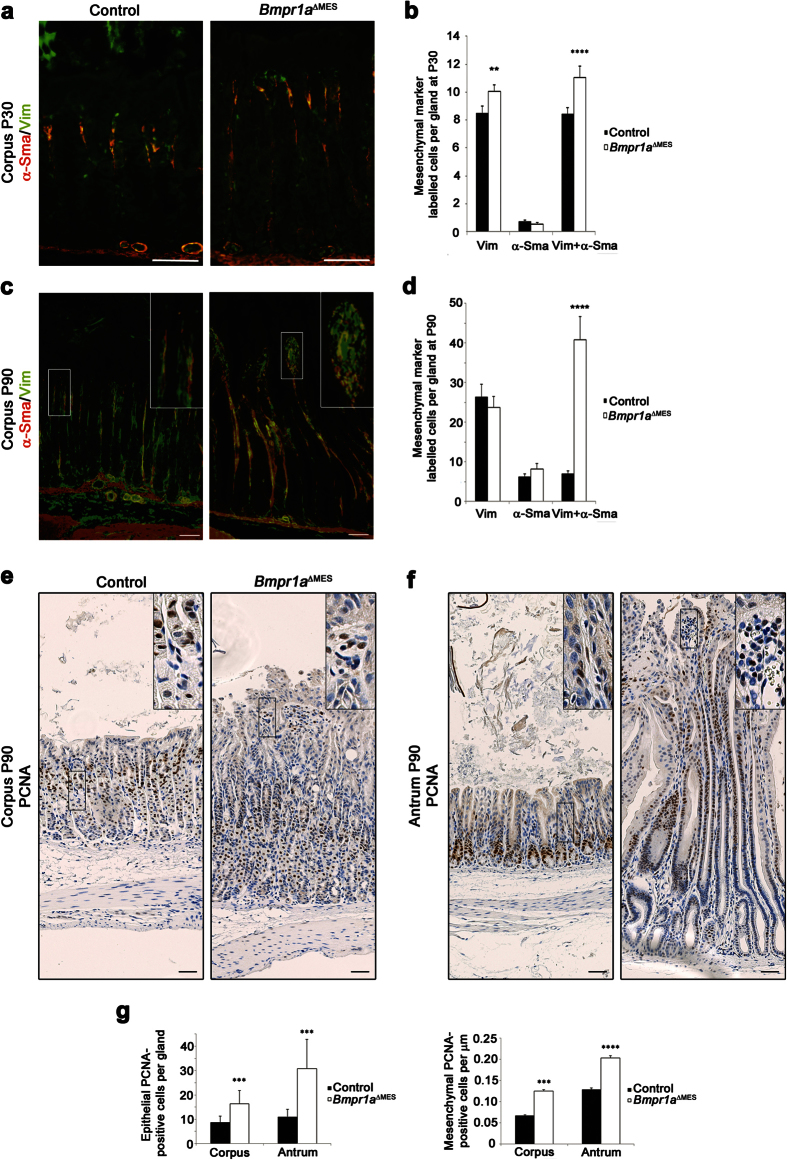
Gastric stromal cellular remodeling in *Bmpr1a*^ΔMES^. **(a**,**c)** Co-staining of vimentin (green staining, stains fibroblasts and activated-fibroblasts) and α-Sma (red staining, stains muscle cells and activated-fibroblasts) was performed in **(a)** 30-day-old and **(c)** 90-day-old mutant and control stomachs. An increased number of activated-fibroblasts (yellow staining) can be observed in mutant mice when compared to controls. **(b**,**d)** Cell counts of double-stained cells relative to single-stained cells revealed an alteration in the ratio of activated-fibroblasts in mutant mice when compared to controls at both **(b)** 30 and **(d)** 90-days. **(e)** Proliferation assays with PCNA staining in 90-day-old control animals showed that proliferation occurred in the isthmus region of the corpus epithelium and in a limited number of cells in the mesenchymal compartment. *Bmpr1a*^ΔMES^ mice showed a significant increase in the number of proliferating cells in the mesenchyme as well as in the corpus epithelium. **(f)** Proliferation assays in 90-day-old control antrum showed that proliferation occurred at the bottom of the gland in the epithelium and in a limited number of cells in the mesenchymal compartment. *Bmpr1a*^ΔMES^ mice showed a significant increase in the number of proliferating cells in the mesenchyme as well as in the antrum epithelium. **(g)** Cell counts of PCNA-stained cells showed a significant increase in the number of proliferating cells in the epithelium of the corpus and antrum glands and increase in the number of mesenchymal proliferative cells in both corpus and antrum. Scale bar: 50 μm. Two-way ANOVA. **p < 0.01; ***p < 0.001; ****p < 0.0001.

Activated-fibroblasts, are a unique group of smooth-muscle-like fibroblasts found in the *lamina propria* of the gut which interact directly with the epithelial cells^11^. The activated-fibroblasts are distinguishable from mesenchymal fibroblasts by their co-expression of vimentin and α-Sma whereas the latter express only vimentin^11^. Stromal remodeling is highlighted by the transdifferentiation of stromal fibroblasts into activated-fibroblasts^37^. We next analyzed whether the loss of Bmp signaling in gastric mesenchymal cells could lead to stromal transdifferentiation in mutant mice by means of co-immunostaining experiments using vimentin and α-Sma. Cell counts of double-stained cells over single-stained cells revealed an increase in the number of fibroblasts (18% increase) and of activated-fibroblasts (31.4% increase) in 30-days-old mutant mice when compared to controls (Fig. 2a,b). Numbers of activated-fibroblasts are increased by 480% by 90-days in mutant mice (Fig. 2c,d). Proliferation assays with PCNA staining in control animals showed that proliferation occurred in the epithelium in the isthmus region of the corpus (Fig. 2e) and at the bottom of the gland in the antrum (Fig. 2f). A limited number of proliferating cells was found in the mesenchymal compartment of the corpus (Fig. 2e) as well as the antrum (Fig. 2f). *Bmpr1a*^ΔMES^ mice showed a significant increase in the number of proliferating cells in the epithelium of the corpus (1.87-fold) and antrum (2.79-fold) glands as well as a significant increase in number of proliferative mesenchymal cells in both corpus (1.85-fold) and antrum (1.57-fold) mesenchyme (Fig. 2g).

### Loss of Bmp signaling in the mesenchyme leads to oxyntic atrophy and impacts gastric epithelial functions

**Figure 3:**
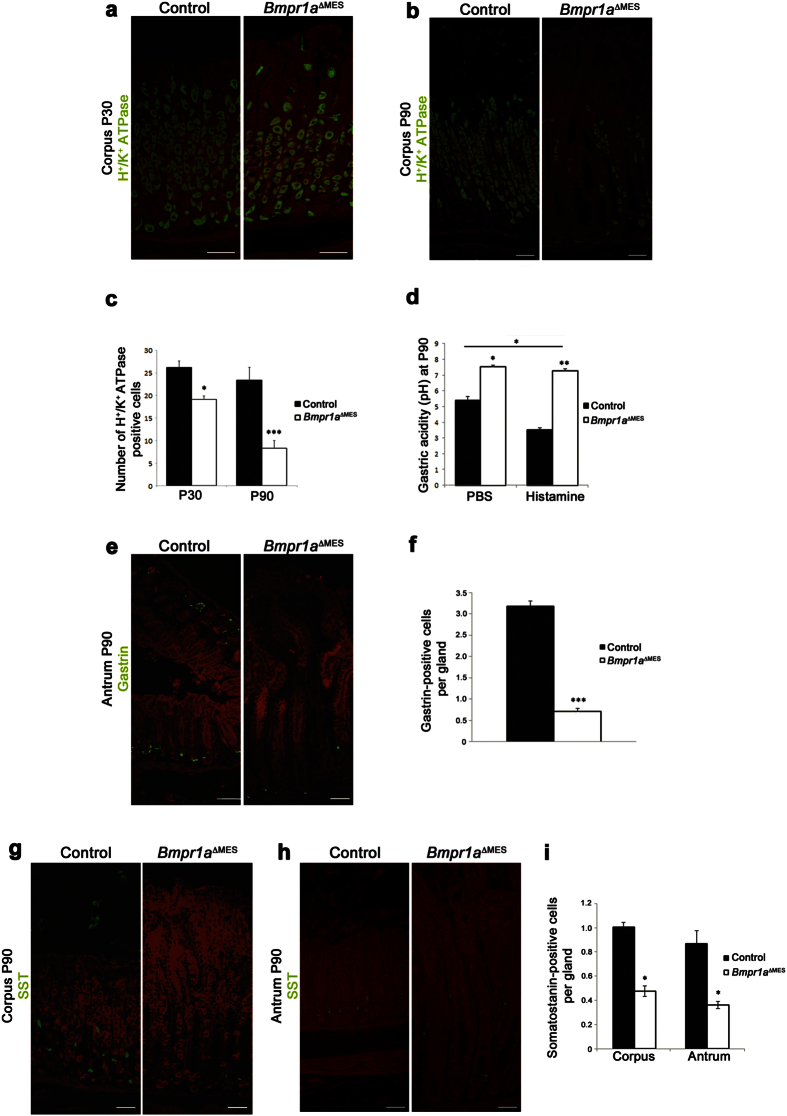
Loss of mesenchymal Bmp signaling leads to gastric hypochlorhydria and modulates gastrin and somatostatin cell populations. (**a**) Proton pump staining associated with parietal cells performed on mutant and control stomachs showed a decrease in the number of parietal cells in 30-day-old mutant stomach (**b**) with a noticeable loss by 90 days. (**c**) Statistical analysis of the number of H^+^/K^+^-ATPase positive cells revealed a significant decrease in parietal cells of the gland units in 30-day-old *Bmpr1a*^ΔMES^ mice and in 90-day-old *Bmpr1a*^ΔMES^ mice (n = 5). (**d**) Measurement of basal (PBS) and stimulated (histamine) gastric acid secretion in 16-hour fasted 90-days-old mice showed that basal and histamine-induced intragastric acidity was significantly lower (higher pH) in both conditions in *Bmpr1a*^*Δ*MES^ mice compared to controls (n = 6). (**e**) Staining against gastrin (green staining) showed a decrease in the number of this sub-population of endocrine cells in 90-day-old *Bmpr1a*^ΔMES^ stomachs compared with controls. (**f**) Positive gastrin-labeled cells were counted from the antrum of control and mutant animals and statistical analysis revealed a significant decrease in number of gastrin cells in *Bmpr1a*^ΔMES^ mice (n = 6). (**g**) Staining against somatostatin (green staining) performed on 90-day-old *Bmpr1a*^ΔMES^ and control corpus and (**h**) antrum revealed a decrease in positively-labeled somatostatin cells in mutant mice. (**i**) Statistical analysis also confirmed the significant decrease in the number of somatostatin-positive cells in *Bmpr1a*^ΔMES^ mice (n = 6). Evans Blue served as counterstain for all immunofluorescences (red staining). Scale bar: 50 μm. Two-way ANOVA (**b,e,g**), Mann-Whitney (**c**); *p < 0.05; **p < 0.01; ***p < 0.001.

Parietal cells in the oxyntic glands play a decisive role in gastric function and homeostasis^38^. Immunostaining of H^+^/K^+^-ATPase associated with the proton pump of parietal cells was performed and showed a decrease in number of labeled acid-secreting parietal cells in *Bmpr1a*^ΔMES^ mice compared to control littermates (Fig. 3a,b). Parietal cell counts revealed a significant 1.37-fold decrease in parietal cells in *Bmpr1a*^ΔMES^ mice at 30 days reaching a 2.81-fold decrease at 90 days (Fig. 3c). In 16-hour fasted 90-day-old mice, basal intragastric acidity was significantly lower by 1.24-fold in *Bmpr1a*^ΔMES^ mice (higher pH) compared to controls (Fig. 3d). *Bmpr1a*^ΔMES^ mice were unresponsive to histamine treatment whereas gastric acidity increased (drop in pH) in histamine-treated control animals. Gastric acid secretion is regulated by endocrine signals through the gastrin-histamine messenger pathway, among others^38^. Morphometric analysis of immunostained stomach pyloric antrums surprisingly revealed a significant decrease in the number of gastrin-expressing cells in *Bmpr1a*^ΔMES^ mice starting at 30 days (2.1-fold) and progressing by 90 days (4.5-fold) compared to controls (Fig. 3e,f).

Analysis in both corpus (Fig. 3g) and antrum (Fig. 3h) also revealed a significant decrease in the number of somatostatin-positive cells (2.1-fold and 2.4-fold, respectively) in 90-days old *Bmpr1a*^ΔMES^ mice compared to controls (Fig. 3i). A significant decrease in somatostatin-positive cells (1.9-fold) was found only in the mutant antrum not in the corpus at 30-days compared to controls (data not shown).

### Loss of Bmp signaling in the mesenchyme influences the development of Spasmolytic polypeptide-expressing metaplasia and intestinal metaplasia without chronic inflammation

**Figure 4:**
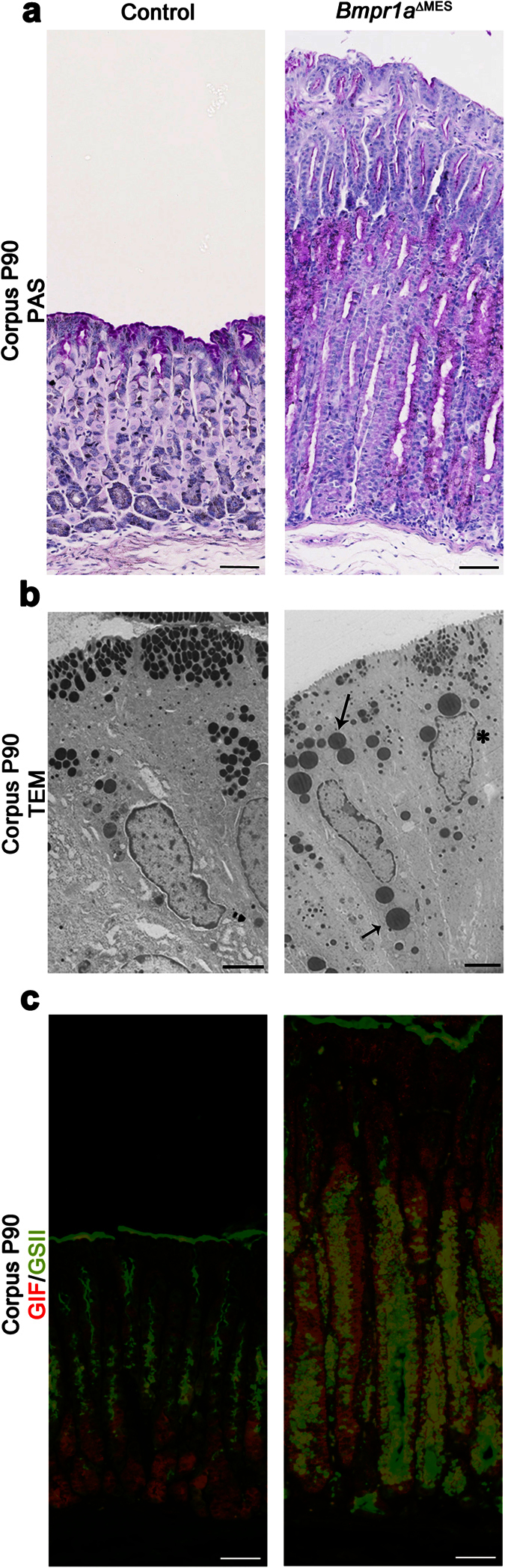
*Bmpr1a*^ΔMES^ mice develop SPEM metaplasia and have impaired mucous secretions. **(a)** Periodic acid–Schiff staining showed an expansion of the surface mucous cell epithelium and revealed a diffuse and lighter staining in 90-day-old *Bmpr1a*^ΔMES^ mice compared with controls. **(b)** Ultrastructural examination of surface mucous cells of oxyntic glands revealed disorganized immature secretory vesicles with no apical orientation (black arrows) and a centrally-located nucleus (black asterisk) in *Bmpr1a*^ΔMES^ mice when compared to controls. **(c)** Immunostaining with gastric intrinsic factor (GIF, in red) allowed the localization of zymogenic cells while lectin labeling for *Grifforia simplifolica* (GSII, in green) allowed localization of mucous neck cells in control gastric gland. Co-staining with GSII and GIF revealed a positive transdifferentiation (yellow staining) of the zymogenic cells in 90-day-old *Bmpr1a*^ΔMES^ mice. Scale bar: 50 μm **(a**,**c)**, 2 μm **(b)**.

*Bmpr1a*^ΔMES^ mice developed a phenotype reminiscent of foveolar hyperplasia with an expansion of the surface mucous cell epithelium into the glandular region of the stomach, as visualized by broader periodic acid–Schiff (PAS) staining when compared to controls (Fig. 4a). In addition, a more diffuse and lighter PAS staining was noted in the mutant mice, suggesting a possible defect in mucous secretion. Transmission electron microscopy revealed ultrastructural changes in secretory vesicles in surface mucous cells of the oxyntic gland in *Bmpr1a*^ΔMES^ mice, as characterized by large disorganized secretory vesicles with no clear apical orientation (Fig. 4b). Surface mucous cells in control mice also displayed a basal nucleus while the nucleus in mutant mice surface mucous cells was centrally located.

We next investigated for the presence of spasmolytic polypeptide-expressing metaplasia (SPEM) in *Bmpr1a*^ΔMES^ gastric mucosa. These SPEM cells are characterized by the presence of mucous neck cell features, including positive reactivity for *Grifforia simplifolica* (GSII) lectin, at the base of the oxyntic glands^38^. Staining for GSII lectin revealed the presence of GSII-positive cells in the zymogenic cell population (immunostained with gastric intrinsic factor) at the base of the glands in *Bmpr1a*^ΔMES^ mice compared to none in the zymogenic cell population in controls (Fig. 4c) suggesting the transdifferentiation of zymogenic cells into SPEM following the loss of Bmp signaling in the mesenchymal compartment.

**Figure 5:**
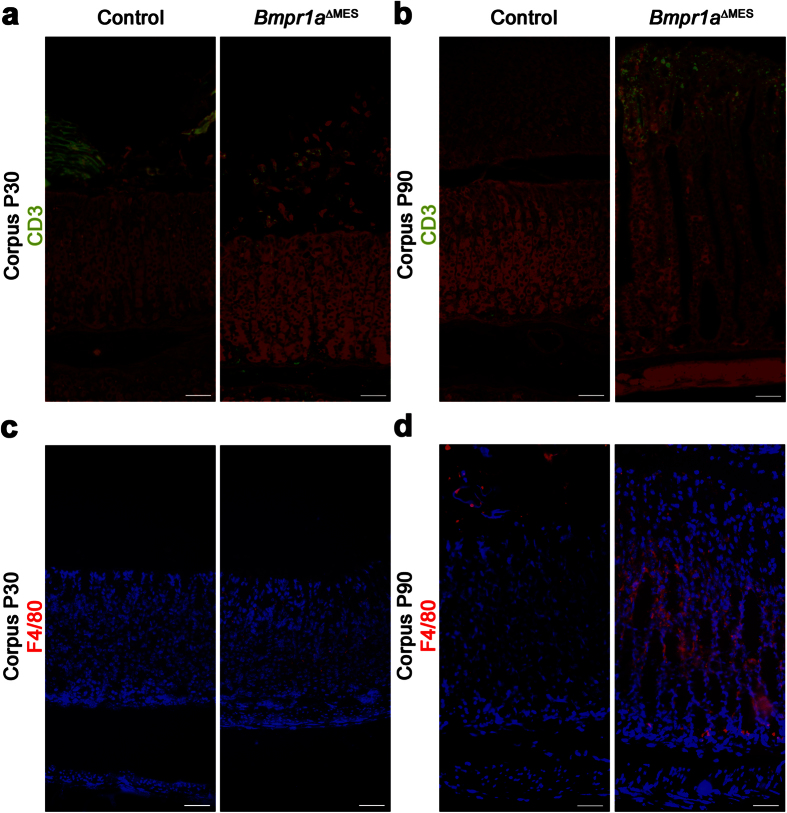
Inflammation does not precede the onset of the phenotypic modifications observed in *Bmpr1a*^ΔMES^ mice. **(a)** Staining against immune cells was performed on the corpus glands of 30-day and 90-day-old mutant and control mice. Staining against CD3 in 30-day-old mice showed no presence of T cells in the mucosa of controls while minimal staining is seen in mutant mice. Cross reactivity (green labelling above the mucosa) with food present in the control stomach lumen is observed. **(b)** Staining against CD3 in 90-day-old mice revealed a prominent presence of T cells in the mucosa of *Bmpr1a*^ΔMES^ mice whereas T cell still remained absent in control mice. Evans Blue served as counterstain (red staining). **(c)** Staining against F4/80 in 30-day-old mice showed no presence of macrophages in the mucosa of control mice where minimal staining is observed in mutants. **(d)** Staining against F4/80 in 90-day-old mice revealed a marked of presence of macrophages in stromal tissue of *Bmpr1a*^ΔMES^ mice while macrophage staining was not observed in control mice. Scale bar: 50 μm.

The above results revealed parietal cell atrophy as well as a possible mucous barrier dysfunction that could lead to increased proliferation of microorganisms in the stomach lumen, thereby intensifying their contact with the epithelium. Such bacterial deregulation could result in chronic inflammation of the gastric mucosa. Accordingly, it is widely recognized that gastric metaplasia precedes neoplastic transformation of the stomach in response to chronic inflammation^39^. Analyses for the atypical presence of T lymphocytes (CD3) and macrophages (F4/80) revealed the absence of these immune cells from the gastric mucosa in control 30-day-old mice (Fig. 5a,c). Presence of a subtle staining for both cell types was found in 30-day-old mutants (Fig. 5a,c). A significant presence of T lymphocytes and macrophages was found once SPEM was already established at 90-days compared to controls (Fig. 5b,d). These results suggest that inflammation did not initiate SPEM in *Bmpr1a*^ΔMES^ gastric mucosa but was rather concomitant with the onset of the phenotypic alterations observed.

**Figure 6:**
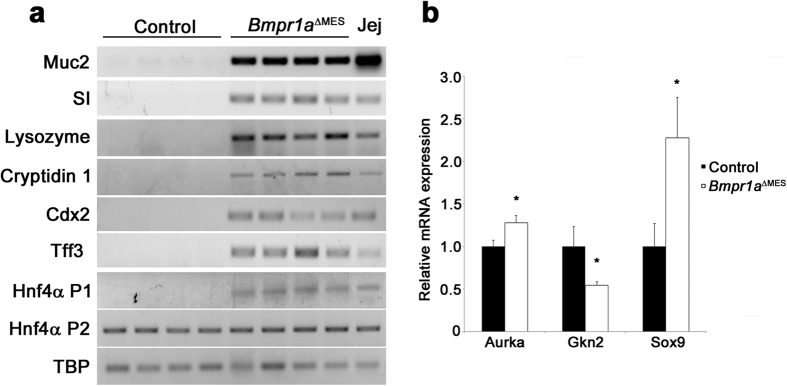
*Bmpr1a*^ΔMES^ mice develop intestinal metaplasia. **(a)** Semi-quantitative RT-PCR analysis from corpus mucosa extract for intestinal goblet, absorptive and Paneth cell markers as well as HNF4α isoforms revealed expression for Muc2, sucrase-isomaltase (SI), lysozyme, cryptidin 1 Cdx2, Tff3, and P1 promoter-driven HNF4α isoforms in P90 gastric mucosa of *Bmpr1a*^ΔMES^ mice whereas no expression of these mRNAs was found in normal stomach (n = 4). P2 promoter-driven HNF4α isoform mRNA was found to be ubiquitously expressed in normal and *Bmpr1a*^ΔMES^ gastric mucosa. Jejunum mucosa served as a positive control. TBP was used as a reference gene. **(b)** Real time qPCR analysis of early gastric cancer genes expression were found to be deregulated in *Bmpr1a*^ΔMES^ mice. Mutant P90 gastric mucosa displayed a significant increase in Aurka and Sox9 mRNA as well as a significant decrease in Gkn2 mRNA expression levels compared with control mice (n = 6). Mann-Whitney; *p < 0.05. Jej, Jejunum.

Intestinal metaplasia (IM) is a recognized premalignant condition of the stomach^38^ and is frequently found, even with early gastric cancer. Stomachs presenting IM lesions display aberrant expression of intestinal markers^38^ such as Muc2 found in intestinal goblet cells, sucrase-isomaltase expressed by absorptive cells and lysozyme associated with Paneth cells^38^. RT-PCR analyses from whole corpus extract support the presence of Muc2, an intestinal goblet cell mucin, sucrase-isomaltase, an intestinal absorptive cells digestive enzyme as well as lysozyme and cryptidin 1, both Paneth cells antimicrobial products, in the *Bmpr1a*^ΔMES^ stomach whereas no expression of these intestinal markers were found in the normal stomach (Fig. 6a). Cdx2 is a transcription factor that regulates the differentiation of intestinal epithelial cells^40^. Its normal expression is strictly confined to the small intestine and colon. Aberrant expression of Cdx2 in the stomach is an important marker for the pathogenesis of IM^41^. RT-PCR analysis confirmed the presence of Cdx2 expression in the *Bmpr1a*^ΔMES^ stomach whereas no expression was found in the normal stomach (Fig. 6a). Trefoil factors are secretory peptides that play a role in mucosal protection. Gastric epithelial cells express Tff1 and Tff2 whereas Tff3 is expressed by intestinal and colonic goblet cells^42^. In IM, Tff3 expression is found to be upregulated by Cdx2 expression^43^. Again, RT-PCR analysis confirmed the presence of Tff3 expression in the *Bmpr1a*^ΔMES^ stomach whereas no expression was found in the normal stomach (Fig. 6a). Hepatocyte nuclear factor-4*α* (HNF4*α*) is a key regulator of several genes involved in glucose and fatty acid metabolism as well as in the pathogenesis of human cancer^44^,^45^. HNF4*α* exists in multiple isoforms generated by alternate P1 and P2 promoter usage and splicing. P1 promoter-driven HNF4α is expressed in hepatocytes, small intestine, colon, kidney and epididymis but is absent in the normal stomach while P2 promoter-driven HNF4*α* is expressed in bile duct, pancreas, stomach, small intestine, colon and epididymis^44^,^45^. However, an altered expression pattern of promoter-driven HNF4*α* is observed in gastric carcinomas where the diseased gastric mucosa expresses P1 promoter-driven HNF4α as opposed to the normally-expressed P2 promoter-driven HNF4*α*^44^. RT-PCR was performed to investigate the presence of the various Hnf4α promoter isoforms in the stomach of both mutant and control. Expression of P1 promoter isoform was found in the *Bmpr1a*^ΔMES^ stomach whereas this isoform was not expressed in the normal stomach (Fig. 6a). No modification in the expression pattern of the P2 promoter isoform was observed between control and mutant stomachs. Lastly, over the years, deregulation in specific markers associated with a precancerous gastric mucosa such as the cancer susceptibility gene *AURKA*^46^, the tumor suppressor gene *GNK2*^47^ or the aberrant expression of hindgut gene *SOX9*^48^,^49^, have been identified. To determine whether these various markers were affected by a loss of mesenchymal Bmp signaling, their respective expression levels in both mutant and wild-type animals were analyzed by qPCR. A significant 1.27-fold and 2.28-fold increase in Aurka and Sox9 mRNA as well as a 1.84-fold significant decrease in Gkn2 mRNA expression levels in mutant gastric mucosa were observed comparatively to controls (Fig. 6b).

### Bmp-deficient gastric mesenchyme leads to ECM remodeling and the development of a reactive mesenchyme expressing Cancer-Associated-Fibroblasts-linked markers

**Figure 7:**
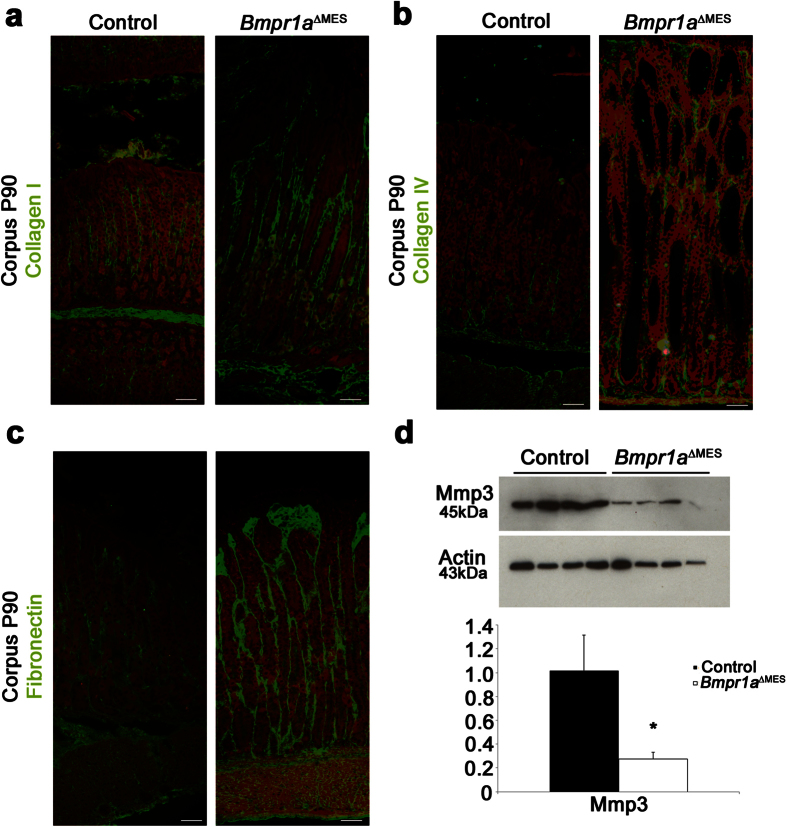
Bmp-deficient activated-fibroblasts lead to gastric ECM remodeling. Immunostaining (in green) against basement membrane **(a)** collagen-I, **(b)** collagen-IV and **(c)** fibronectin was performed in the corpus gland of 90-day-old control and mutant mice. Collagen-I, collagen-IV and fibronectin deposition were increased in *Bmpr1a*^ΔMES^ mice compared to controls. Evans Blue served as counterstain for all immunofluorescences (red staining). **(d)** Western blot analyses in 90-day-old mice revealed a decrease in matrix metalloproteinase 3 (Mmp-3) in *Bmpr1a*^ΔMES^ mice compared to controls. Actin served as loading control. Densitometry analysis of exposed films using ImageJ revealed a significant decrease in Mmp-3 expression compared to controls (n = 4). Mann-Whitney; *p < 0.05. Scale bar: 50 μm.

A pathological reactive mesenchyme is frequently populated by an abnormal number of activated-fibroblasts. Given that such reactive mesenchyme leads to the production of a toxic microenvironment with deregulated levels/types of cytokines, growth factors and massive remodeling of the ECM thus promoting the pathological state in the epithelium^14^,^33^,^34^,^37^, we next analyzed the potential alteration of ECM composition in *Bmpr1a*^*ΔMES*^ mice. Immunostaining against collagen-I, collagen-IV and fibronectin confirmed the increased deposition of these ECM proteins in 90-days-old *Bmpr1a*^ΔMES^ mice compared to controls (Fig. 7a–c, respectively). Lastly, Western blot analyses on corpus from whole stomach extract revealed a significant 3.72-fold down-modulation in stromelysin-1 (Mmp3), an important matrix-remodeling endopeptidase (Fig. 7d).

Considering that the reactive mesenchyme is deeply involved in the production of secreted soluble factors that potentially affect tumor-promoting pathways such as the Bmp, HGF and Wnt pathways^50^, the expression levels of factors involved in these pathways were analyzed by qPCR on corpus from whole stomach extract of 30-days-old *Bmpr1a*^*ΔMES*^ and control mice. No modulations were observed in Bmp 2, 4, and 7 mRNA expression levels; however a sharp 9.34 fold decrease in noggin mRNA expression levels was notably observed in *Bmpr1a*^*ΔMES*^ compared to control mice (Table 1). The overall ratio of inhibitor modulation suggests a deregulation in Bmp signaling but without intense variation of epithelial Bmp intracellular signaling as demonstrated by pSmad1-5-8 immunofluorescence in the gastric epithelium (Fig. 1d). A significant 2.54-fold increase in HGF mRNA and a significant 2.34-fold decrease in sFRP1 (Wnt pathway signaling inhibitor) mRNA expression were also found in mutant gastric mucosa compared to controls (Table 1). Moreover, qPCR analysis revealed a significant 3.47-fold increase in FSP1/S100A4, an important Cancer-Associated-Fibroblasts (CAFs) marker^50^ in *Bmpr1a*^*ΔMES*^ compared to control mice (Table 1). The latter, in addition to the above increase in α-Sma stromal expression and deposition of collagen-I as well as fibronectin in mutant mice, all well-known CAFs markers^14^,^50^, strongly suggest a role for mesenchymal Bmp signaling in promoting the establishment of a precancerous reactive mesenchyme.

## Discussion

Until recently, mesenchymal cells were predominantly recognized for their role in sustaining the gastric epithelium rather than for their potential contribution to its pathologies^33^. However, it is now well established that genetic changes in troubled epithelium are insufficient to fully explain disease initiation and progression^31^,^51^. Accordingly, it is beginning to be accepted that cells within the mesenchyme may play a critical role in many gastric disorders^9^,^33^,^52^. The pathological reactive mesenchyme is frequently populated by an abnormal number of activated-fibroblasts, which in turn leads to the production of a toxic microenvironment for the epithelium promoting tumor growth during the earliest stages of neoplasia^14^,^33^,^37^,^50^. In this particular context, mesenchyme remodeling is highlighted by the excessive transdifferentiation of stromal fibroblasts into activated-fibroblasts^37^. Nonetheless, most studies have been unable to identify the exact molecular mechanisms, signaling pathways or genetic mutation(s) responsible for promoting normal activated-fibroblasts to adapt into a reactive mesenchyme. Previous studies using genetic approaches in mice have revealed a role for Bmp signaling in gastric tumorigenesis^24^,^26^,^28^. However, one of the common characteristics of these various mouse models was the impairment of Bmp signaling in all cell layers of the tissue. Conversely, studies from our laboratory revealed that exclusive loss of gastric Bmp signaling in the epithelium was not sufficient to trigger gastric tumorigenesis^6^. Our findings suggested that extra-epithelial Bmp signaling played a greater role in gastric neoplasia initiation than originally suspected. In the present study, we conditionally inactivated *Bmpr1a* in mouse gastric sub-epithelial mesenchyme in order to specify the function of mesenchymal Bmp signaling pathway and its impact on gastric mucosal biology. Using this model, we were able to expose a key role of Bmp signaling in preventing normal gastric activated-fibroblasts to adapt into reactive mesenchyme. In addition, *Bmpr1a*^*Δ*MES^ mice showed the occurrence of spontaneous gastric benign polyps as early as 90-days post-natal. Further analysis confirmed the presence of both IM and SPEM in *Bmpr1a*^*Δ*MES^ mice.

Some aspects of tumor development are akin to processes observed in developing organs^53^. The mesenchyme, and more specifically the activated-fibroblasts, is known to be essential for normal gastrointestinal organ development through its production of cytokines, growth factors and ECM proteins^7^. These various soluble and insoluble factors establish a specific microenvironment guiding a specific response from the epithelium as well as the surrounding stroma. For example, mesodermal Bmp4 is responsible for establishing a particular paracrine signaling potential in the epithelium to promote normal epithelial development along the entire chick gastrointestinal rostro-caudal axis^7^. On the other hand, Bmps also activate mesenchymal signaling cascades allowing mesoderm specification and thereby assigning it new potential for influencing epithelial determination during gastrointestinal development^54^,^55^. While some microenvironments are nurturing for the overlying epithelium, others are favorable for initiation or progression of diseased epithelium. As aforementioned, mutations affecting Bmp signaling are frequently observed in JPS^20^,^21^ as well as in tumor progression in the stomach^22^,^23^ with a manifest overexpansion of the mesenchymal compartment^14^. Moreover, in digestive cancers, such as gastric cancer, the reactive mesenchyme clearly precedes epithelial dysplasia^39^.

The present data confirmed an increase in mesenchymal cell proliferation with an early transdifferentiation process into activated-fibroblasts in *Bmpr1a*^ΔMES^ mice. Our results thus indicate that loss of Bmp signaling in the mesenchyme appears to be a critical threshold event in creating a primed mesenchyme that must occur before the reactive mesenchyme can support the initiation of pre-malignant lesions. Moreover, with the various modifications observed in *Bmpr1a*^ΔMES^ mice such as significant expansion of α-Sma-positive fibroblasts and increased production of collagen-I, fibronectin, HGF and FSP1/S100A4, all known markers indicative of transformation of activated-fibroblasts into CAFs, our current study also revealed that, with time, loss of mesenchymal Bmp signaling is putatively an early event in priming activated-fibroblasts toward the CAFs phenotype. We also observed an important decrease in noggin expression in the mutant mice. This decrease in noggin expression could be explained by the fact that *Bmpr1a*-deficient activated-fibroblasts are devoid of Bmp signaling and, given the need for this signaling pathway, they are thus not predisposed to produce a Bmp pathway antagonist. Though this specific Bmp signaling inhibitor is decreased, it has no particular impact on the epithelial Bmp signaling as demonstrated by pSmad1-5-8 immunofluorescence in the gastric mucosa. Additionally, our data demonstrate that *Bmpr1a*-deficient activated-fibroblasts secreted reduced level of Wnt pathway antagonist sFRP1. Indeed, in gastric cancer, activation of Wnt signaling appears to be a major oncogenic pathway causing tumor development^25^,^56^.

Altogether, deregulation in the expression levels of these numerous soluble and insoluble factors in *Bmpr1a*^*Δ*MES^ mice results in the production of a toxic microenvironment^37^,^57^ thereby incurring increased pressure on the gastric epithelium and promoting epithelial neoplasia initiation. Indeed, the direct impact of the loss of Bmp signaling in the mesenchyme on the epithelium is the development of both types of metaplasia associated with the precancerous stomach. Our results also confirmed the absence of an acute inflammatory event preceding the emergence of these metaplasias. Although there was a clear development of polyps assembled into precancerous lesion in the stomach of *Bmpr1a*^*Δ*MES^ mice, as revealed by histopathological analysis as well as from variations in early gastric cancer-associated genes such as *AURKA*, *GNK2* and *SOX9*, there was no progression toward a more malignant state of the disease. Over the years, it has been well documented that infection of gastric mucosa by *Helicobacter pylori* leads to inflammation-associated carcinogenesis^38^,^39^. Furthermore, using a genetic approach, Oshima *et al*. as well as Takabayashi *et al*. showed that transgenic mice overexpressing noggin (which ablates Bmp signaling in all cell layers of the gastric mucosa) in combination with elevated levels of Prostaglandin E2^28^ or in presence of *H. felis* bacterial infection^27^ (both, an inflammatory trigger), led to the development of more advanced gastric lesions^28^. One could speculate that induction of the inflammatory response in the context of the loss of mesenchymal Bmp signaling could lead to a dramatic cascade of events resulting in increased susceptibility to neoplastic progression. Notwithstanding the latter, our findings nonetheless support that loss of Bmp signaling within the mesenchyme regulates several gastric oncogenic epithelial cell behaviors and functions.

As aforementioned, developmental as well as adult studies in mice have demonstrate the importance of the mesenchyme in instructing the GI epithelium^7^,^8^,^19^,^58^. We believe also that a specific signaling within the stroma along the rostro-caudal axis of the gut could influence differently their respective organs. This can be exemplified by our recent study on the loss of Bmps signaling in the colonic mesenchyme^32^. Some results shown in this paper are similar to the current study. For example, the loss of mesenchymal Bmp signaling in both GI sections leads to an important transdifferentiation of fibroblasts into activated fibroblast, development of polyposis as well as deregulation of the extracellular soluble and insoluble milieu. However, the main difference lies in the composition of that extracellular soluble milieu. In the stomach, we found an early deregulation of the microenvironment in the mutant mouse composed of factors linked with cancer-associated fibroblasts. Also, the impact of the Bmp mesenchymal deletion on the gastric epithelium is of a precancerous nature with early apparition of SPEM and IM and presence of early gastric cancer-associated genes. In the colon, the polyposis phenotype occurred later in life around 9 months to 1 year of age. Although some cancer-associated fibroblasts factors are present, the extracellular milieu has an important inflammatory signature with deregulated cytokines and chemokines. Finally, the pathological changes observed in the colon are similar to those described for the Hamartomatous Polyposis Syndrome in humans, particularly Juvenile Polyposis Syndrome.

In conclusion, the present results validate our proposed concept that loss of the Bmp signaling cascade within mesenchymal cells acts as a trigger in gastric tumorigenesis initiation through the transdifferentiation of activated-fibroblasts leading to the development of a reactive mesenchyme. As such, Bmp signaling might be considered as a potential candidate contributing to the transition of activated-fibroblasts toward the Cancer-Associated-Fibroblasts phenotype.

## Materials and Methods

### Animals

129SvEv-*Bmpr1a*^fx/fx^ mice were provided by Dr. Mishina^35^ and backcrossed with C57BL/6J mice for 20 generations. The C57BL/6J *Foxl1*Cre transgenic line was provided by Dr. Kaestner^36^. For this study, the C57BL/6J-*Bmpr1a*^fx/fx^ mice were first crossed with the C57BL/6 *Foxl1*Cre to generate *Bmpr1a*^fx/+^; *Foxl1*Cre animals which were then crossed with *Bmpr1a*^fx/fx^ mice to produce *Bmpr1a*^fx/fx^;*Foxl1*Cre (*Bmpr1a*^ΔMES^) experimental animals and their controls. Genomic DNA was isolated using the Spin Doctor genomic DNA kit from Gerard Biotech according to the manufacturer’s protocol. All mutations were genotyped according to previously published protocols^35^,^36^. All experiments were approved by the Animal Research Committee of the Faculty of Medicine and Health Sciences of the Université de Sherbrooke (AEC approval number FMSS-114-15). The study followed the standards and policies of the Canadian Council on Animal Care in sciences.

### Mesenchymal enrichment and genomic DNA extraction

Genomic DNA was isolated from enrichment of epithelial and mesenchymal compartments of the stomach of 1-year-old mice by an adaptation of the MatriSperse dissociation method^59^,^60^. Briefly, mice were sacrificed and the stomach was collected. The stomach was opened longitudinally and rinsed with cold PBS. The sections were further cut in 5-mm pieces and incubated in 5 ml of cold MatriSperse (Becton-Dickinson) in 15-ml tubes at 4 °C for 24 h. The epithelial layer was dissociated by gentle manual shaking. The epithelial suspension was collected and discarded. The remaining fraction represented mesenchymal enrichment and was washed with cold PBS. Genomic DNA extraction was performed with the DNeasy Blood and tissue Kit (QIAGEN).

### Cre-dependent loxP allele recombination validation

PCR analysis was used to validate the recombination of *Bmpr1a/*loxP allele in the mesenchymal extract enrichment. Cre dependent recombination between the two loxP sites was visualized by PCR amplification of a 180-bp fragment using specific fx1 (GGTTTGGATCTTAACCTTAGG) and fx4 (TGGCTACAATTTGTCTCATGC) primers^35^.

### Tissue collection, RNA extraction and gene expression analysis

Total RNA was isolated from the corpus of the whole stomach and processed using the Totally RNA extraction kit (Ambion), RT-PCR and qPCR were performed as described previously^6^,^17^. Quantitative real-time PCR was performed by the Plateforme RNomique de l’Université de Sherbrooke; primer sequences are available upon request.

### Tissue preparation, histological and immunofluorescence staining

Stomachs from 30- to 90-day-old *Bmpr1a*^ΔMES^ and control littermates were fixed, sectioned and stained as previously described^6^,^17^ or according to the manufacturer’s protocol (395B-1KT Sigma-Aldrich). Immunostainings were performed as previously described^6^,^59^. The following antibodies were used at the indicated dilutions: anti-PCNA (1:10,000, Abcam), anti-Bmpr1a (1:300, Abcam), anti-pSmad1-5-8 Ser463-465 (1:500, Santa Cruz), 488 Alexa Fluor Lectin GSII (1:200, Invitrogen) anti-H+/K+-ATPase (1:2, MBL), anti-GIF (1:75, Abnova), anti-somatostatin (1:100, Santa Cruz), anti-gastrin (1:100, Chemicon), anti-CD3 (1:200, Dako), anti-F4/80 (1:100, eBioscience), anti- α-Sma clone 1A4 (1:5000, Sigma), anti-Vimentin^XP^ D21H3 (1:100, Cell Signaling), anti-fibronectin (1:1000, Millipore), anti-collagen-I (1:200, Thermo Scientific), anti-collagen-IV (1:200, Chemicon International) and FITC-conjugated anti-rabbit IgG (1:300, Vector), FITC-conjugated anti-goat IgG (1:300, Vector) and Alexa 568-conjugated anti-mouse (1:400, Invitrogen). For immunofluorescence, images were captured on a Leica DMLB2 microscope using a Leica DC300 camera except for α-Sma/pSmad1-5-8 immunostaining and α-Sma/Bmpr1a where a Confocal Zeiss LSM700 along with Zen Blue software was used. For IHC, images were captured on a Nanozoomer Digital slides Scanner (Hamamatsu).

### Protein extraction and Western blot analysis

Total proteins were isolated from corpus of the whole stomach of 90 day-old *Bmpr1a*^ΔMES^ and control mice with RIPA buffer (50 mM Tris pH 7.4, 150 mM NaCl, 1% NP40, 0.5% Triton X-100, 1 mM EDTA, 0.2% SDS, 0.5% Na-deoxycholate) containing protease and phosphatase inhibitors^3^,^11^. Western blotting was performed as previously described^6^,^17^. The following affinity-purified antibodies were used: anti-Mmp3 monoclonal antibody (1:3000, clone: SL-1 111C4, Oncogene), Actin (1:10 000, clone C4, Millipore) HRP-anti-mouse (1:6000, Cell Signaling) and HRP-anti-rabbit (1:6000, Cell Signaling). For densitometry analyses, exposed Western blot films were scanned and images were analyzed using ImageJ (Rasband, WS, ImageJ, US National Institutes of Health, Bethesda, Maryland, USA).

### Electron microscopy

Portions of mouse stomach corpus were rinsed with PBS, prefixed for 15 min with a 1:1 mixture of culture medium (Dulbecco’s modified Eagle’s medium) and freshly prepared 2.8% glutaraldehyde in cacodylate buffer (0.1 M cacodylate and 7.5% sucrose), then fixed for 30 min with 2.8% glutaraldehyde at room temperature. After two rinses, specimens were postfixed for 1 h with 2% osmium tetroxide in cacodylate buffer. The tissues were then dehydrated using graded ethanol concentrations (40, 70, 90, 95, and 100%, three times each) and coated twice for 3 h with a thin layer of Araldite 502 resin (for ethanol substitution). Finally the resin was allowed to polymerize at 60 °C for 48 h. The specimens were detached from the plastic vessels, inverted in embedding molds, immersed in Araldite 502, and polymerized at 60 °C for 48 h. Ultramicrotome-prepared thin sections were contrasted with lead citrate and uranyl acetate, then observed on a Jeol 100 CX transmission electron microscope. All reagents were purchased from Electron Microscopy Sciences (Cedarlane, Hornby, ON, Canada)^59^.

### Measurement of basal and stimulated gastric acid secretion

Gastric acidity values were determined from 16-h fasted control and *Bmpr1a*^ΔMES^ mice as previously described^6^.

### Quantification of cell number and statistical analyses

Positively-stained cells for PCNA, α-Sma, vimentin, H^+^/K^+^-ATPase, somatostatin, gastrin were counted per glandular axis. The total number of nuclei was quantified using DAPI staining. Two-way ANOVA or Mann-Whitney was used for statistical analysis of the percentage of positive cells per glandular axis. All cell count analyses were performed using continuous sections from low-powered fields of well-oriented glandular cross-sections in a blinded manner on an average of 10 independent fields per animal (n = 4). Data are expressed as mean ± SEM or mean ± SD. All statistical analyses were carried out using Graph Pad Prism 6 (Graph Pad Inc, San Jose, CA). Differences were considered significant at a *p* value of <0.05.

## Additional Information

**How to cite this article**: Roy, S. A. B. *et al*. Loss of mesenchymal bone morphogenetic protein signaling leads to development of reactive stroma and initiation of the gastric neoplastic cascade. *Sci. Rep.* **6**, 32759; doi: 10.1038/srep32759 (2016).

## Figures and Tables

**Table 1:** CAF markers and soluble factors gene expression changes in *Bmpr1a*^ΔMES^ mice.

Gene Description	Gene Symbol	Fold	*P* Value
Wnt Pathway			
Secreted frizzled-related protein 1	*sFrp1*	−2.34	0.038
Dickkop homolog 1	*Dkk*	−2.28	NS
Wingless-type MMTV integration site family member 5a	*Wnt5a*	1.08	NS
Bmp pathway			
Bone morphogenetic protein 2	*Bmp2*	−1.25	NS
Bone morphogenetic protein 4	*Bmp4*	−1.21	NS
Bone morphogenetic protein 7	*Bmp7*	−1.02	NS
Noggin	*Nog*	−9.34	0.012
Chordin	*Chrd*	−3.82	0.017
Growth Factor			
Hepatocyte growth factor	*Hgf*	2.54	0.017
Insulin-like growth factor	*Igf*	−1.46	NS
CAF Markers			
Fibroblast surface protein	*Fsp1/S100A4*	3.47	0.002

Fold change represents the ratio of mean expression values (control/mutant) derived for qPCR data. Negative values indicate reduction in *Bmpr1a*^ΔMES^ mice. NS = nonsignificant fold change (Mann-Whitney U test).
